# Income disparities in mental health: investigating the role of food insecurity by disability status

**DOI:** 10.1017/S1368980022002063

**Published:** 2023-04

**Authors:** Meredith R Williams, D Phuong Do

**Affiliations:** Joseph J. Zilber School of Public Health, University of Wisconsin-Milwaukee, P.O. Box 413, Milwaukee, WI 53201-0413, USA

**Keywords:** Disability, Income, Food security, Psychological distress, Mental health

## Abstract

**Objective:**

To investigate whether food insecurity helps explain the association between income and psychological distress and if its role differs by disability status.

**Design:**

Using 2011–2017 National Health Interview Survey cross-sectional data (*n* 102 543), we conducted linear regression models, fully interacted with disability status, to estimate the association between income-to-poverty ratio (IPR) (<1, 1–<2, 2–<4, ≥4) and psychological distress (Kessler 6 (K6) Scale, range: 0–24). Base models adjusted for socio-demographic factors. We then added food security (secure, low and very low), interacted with disability, and conducted post-estimation adjusted Wald tests.

**Setting:**

USA.

**Participants:**

Nationally representative sample of non-institutionalised adults 18 years and older.

**Results:**

The association between income and psychological distress was stronger for people with disabilities. Compared to those in the highest income category (IPR ≥4), poor individuals (IPR < 1) with and without disabilities scored 2·10 (95 % CI (1·74, 2·46)) and 0·81 (95 % CI (0·69, 0·93)) points higher on the K6 Scale, respectively. Accounting for food insecurity reduced the estimated income disparity in psychological distress significantly more among individuals with disabilities (0·96 points or 46 %) than without disabilities (0·34 points or 42 %), decreasing the difference in the income disparity between those with and without disabilities by 48 % (0·62 points). Further, food insecurity more strongly predicted psychological distress for individuals with disabilities independent of socio-economic disadvantage.

**Conclusions:**

Food insecurity plays a more important role in shaping patterns of psychological distress for people with disabilities, explaining more of the association between income and psychological distress among those with than without disabilities. Improving food security may reduce mental health disparities.

The adverse association between low income and psychological distress has been established across time and around the world^(1,2)^. In longitudinal studies, individuals who experience decreases in income or transition into poverty report subsequent declines in mental health^(1,2)^. Recent evidence further indicates that living in poverty predicts psychological distress more strongly among people with disabilities than those without disabilities^(3)^. This finding is particularly concerning given that people with disabilities are over twice as likely as those without disabilities to report incomes below the poverty threshold^(3)^. Moreover, research suggests that even small increases in levels of psychological distress can have serious consequences for health, predicting increased odds of engaging in health-harming behaviours, developing preventable chronic diseases and dying prematurely^(4–6)^. Despite these risks, factors that might explain the stronger adverse association between poverty and poor mental health for people with disabilities have yet to be explored.

Most studies investigating potential mediators in the relationship between poverty and mental health among the general population have focussed on psychosocial factors. Results from extant research are inconclusive, with some studies finding that financial hardship contributes to poorer mental health by reducing individuals’ sense of agency, control and self-esteem, and others finding no evidence that psychological factors play a role in the relationship between income and mental health^(7)^. Fewer studies have examined whether material hardship explains the association between poverty and psychological distress. These studies typically rely upon a composite index of material deprivation that combines multiple measures (e.g. food insecurity, transportation, housing, telephone and Internet). Findings indicate that material hardships explain much, but not all, of the association between poverty and poor mental health among pregnant women^(8)^, mothers^(9)^ and low-income service workers^(10)^. For instance, one study found that increases in material hardships explained 43 % of this adverse association^(10)^. Another demonstrated that material deprivation explained 32 % of the observed decline in mental health among individuals who developed a disability, compared to only 5 % for behavioural and 1 % for psychosocial factors^(11)^.

The role of one specific type of material deprivation, food insecurity, has seldom been examined in isolation. Only two studies, to the best of our knowledge, have investigated the potential mediating role of food insecurity in the adverse association between poverty and poor mental health. In a cross-sectional study of Singaporean adults^(12)^, food insecurity fully explained the detrimental relationship between poverty and psychological distress. In another study, a poverty-alleviation intervention was delivered to ultra-poor women in Bangladesh^(13)^. The intervention reduced distress among participants, with improved food security explaining 48 % of this decrease^(13)^.

The lack of research investigating the role of food insecurity in the adverse association between poverty and psychological distress is striking given that food insecurity is more strongly associated with a family’s income-to-poverty ratio (IPR) than crime, housing issues, neighbourhood problems, access to consumer durables, healthcare and difficulty meeting basic needs, and is correlated with even very small and short spells of poverty^(14)^. Moreover, a large body of cross-sectional and longitudinal studies, systematic reviews and meta-analyses demonstrates that as food security decreases, scores on self-rated mental health, depression and psychological distress scales worsen^(15–18)^.

Those living in food insecure households often consume lower-quality, less-balanced and nutrient-poor diets, all of which have been shown to directly contribute to the development of depression, anxiety and distress^(19)^. In the classic Minnesota Starvation Experiment^(20)^, formerly healthy young men subjected to semi-starvation displayed symptoms of depression, irritability, anxiety and impaired cognition. As participants entered the rehabilitation phase of the experiment, decreases in depression scores correlated strongly with calories consumed, with those who received diets containing the most calories experiencing the most rapid improvements in depression^(20)^. Mental health can also be compromised by the stress and worry that accompanies food insecurity. Food insecure individuals report feeling stress, shame, guilt, social alienation and powerlessness due to their difficulty obtaining adequate food in a socially acceptable manner^(16)^.

Theory further suggests that the influence of financial and material deprivation on health outcomes may be magnified for people with disabilities. The World Health Organisation’s^(21)^ International Classification of Functioning, Disability and Health (ICF) proposes that an individual’s social and environmental circumstances, including income and food security, dynamically interact with their underlying health conditions, leading to greater disability and worse health outcomes than would be expected based on their underlying conditions alone. This framework is consistent with extant research demonstrating that low income more strongly predicts poor mental health among people with disabilities than among those without disabilities^(3)^. It is possible that food insecurity also plays a disproportionate role in explaining the adverse association between low income and poor mental health for people with disabilities.

For instance, even within the same level of income, individuals with disabilities are more likely to experience food insecurity^(22–27)^, report higher competing expenses and experience greater unmet needs for other goods and services than those without disabilities^(28)^. Many people with disabilities require expensive, high-quality and speciality dietary items to effectively manage their medical conditions^(25)^, the absence of which may aggravate their health and exacerbate stress and worry. While one study found that food insecurity predicted poor mental health similarly among those with and without disabilities^(26)^, no study, to the best of our knowledge, has investigated the extent to which food insecurity helps explain the burden of poverty on mental health or whether the magnitude of its mediating role differs by disability status.

To address this gap, we investigated whether food insecurity helps to explain the deleterious association between low income and psychological distress in the US population and whether its role differs by disability status. Consistent with the ICF^(21)^, we hypothesised that food insecurity may explain more of the income-psychological distress gradient and play a stronger role in shaping patterns of poor mental health among people with disabilities than those without disabilities, as illustrated in Fig. 1.

**Fig. 1:**
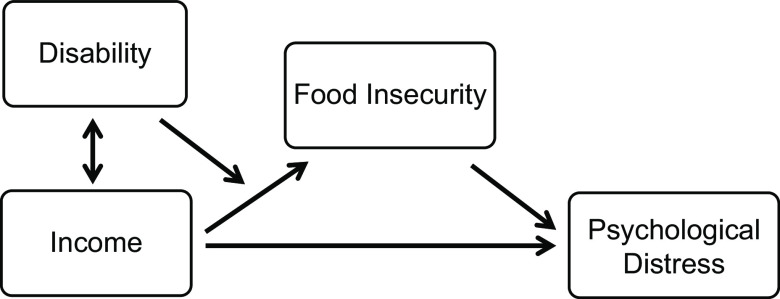
Illustration of the hypothesised relationships between disability status, income, food insecurity and psychological distress. The double-headed arrow between disability and income reflects the bidirectional relationship between disability and income. The unidirectional arrow intersecting the income–food insecurity pathway indicates the potential moderating influence of disability on the income–food insecurity–psychological distress pathway

## Methods

### Data

We analysed data from the Integrated Public Use Microdata Series version of the 2011–2017 National Health Interview Survey (NHIS)^(29)^, the latest years in which both food security measures and the full set of disability questions outlined in the Department of Health and Human Services (2011) data collection standard are available. The NHIS is an annual, cross-sectional survey that collects nationally representative data on the health and socio-demographic characteristics of non-institutionalised US civilians. We restricted all analyses to adults aged 18 and older for whom there were no missing values on our variables of interest. Our analytical sample included 102 598 respondents, of whom 20 952 had a disability.

### Measures

#### Outcome

Our outcome of interest was non-specific psychological distress, measured by the Kessler 6 (K6) Scale. The K6 (Cronbach’s α = 0·89) is a screening tool used in large, nationally representative surveys to assess the prevalence and frequency of six psychological symptoms indicative, but not diagnostic, of non-specific psychological distress^(30,31)^. The K6 Scale is composed of questions about the frequency of psychological distress symptoms experienced over the past 30 d, including feeling: (1) sad; (2) nervous; (3) restless and fidgety; (4) hopeless; (5) worthless and (6) that daily activities require too much effort. Responses to each item were assigned a frequency-based score, ranging from 0 for “none” to 4 for “all of the time.” For each respondent, scores from all K6 items were summed, resulting in a continuous K6 score ranging from 0 (i.e. no psychological distress) to 24 (i.e. highest possible level of psychological distress).

Studies of psychological distress often exclude participants with mental illness (e.g. clinically diagnosed schizophrenia, anxiety, depression) in an effort to minimise confounding^(31,32)^. However, psychological distress and mental illness, although related, are separate constructs^(31)^. Accordingly, social and material disadvantages predicting greater levels of psychological distress among people with preexisting mental health conditions^(31,32)^. In light of this evidence, we did not exclude respondents with mental illness from the current study.

#### Key variables of interest

Our three key variables of interest were income, disability status and food security. We used IPR to measure household income, calculated as the ratio of total combined family income to the poverty threshold as defined by the US Census Bureau. IPR was categorised according to four levels: <1, 1 to <2, 2 to <4, or ≥4. We specified disability status as a binary indicator based on answering yes to at least one of six NHIS questions described in the Department of Health and Human Services^(33)^ data collection standard. These questions are intended to assess whether respondents are deaf or have ‘serious difficulty hearing,’ blind or have ‘serious difficulty seeing’ with glasses, have ‘serious difficulty concentrating, remembering, or making decisions,’ ‘serious difficulty walking or climbing stairs,’ difficulty with dressing or bathing and difficulties running errands or attending medical appointments alone^(33)^.

Food security was assessed using the US Adult Food Security Survey Module^(34)^, a 10-item, three-stage screening tool designed to measure household food security over the past 30 d. Points were assigned according to the respondent’s answers to a series of questions regarding the severity of food shortage, if any. In the first stage, respondents are asked whether three statements were ‘often’, ‘sometimes’, or ‘never’ true because their households did not have enough money for food: (1) they worried they would run out of food; (2) food they bought did not last and (3) they could not eat balanced meals. In stage two, those who indicated any food scarcity (i.e. ‘sometimes’ or ‘often’ true) were asked if, because they did not have enough money for food, they: (1) ate less than they felt they should; (2) did not eat when hungry; (3) lost weight and (4) ate smaller meals or skipped meals and, if so, the number of days they did so. In the final and third stage, respondents were asked if there was ever a full day during which they did not eat because there was not enough money for food and, if so, the number of days this occurred. The scores from each stage of the module were summed, resulting in a total raw score ranging from 0 to 10. Given that even very low levels of food insecurity increase the risk of poor health outcomes, Gundersen and colleagues^(35)^ suggest that relying on binary indicators of food security may underestimate the impact of food insecurity on mental health and mask differences in the strength of that relationship by characteristics such as disability status. Thus, consistent with USDA’s recommendations for classification^(34)^, food security was categorised into three levels: food secure (raw score: 0–1), low food security (raw score: 2–5) and very low food security (raw score: 6–10).

#### Covariates

Analyses adjusted for educational attainment (less than high school, high school or general educational development, some college, bachelor’s degree or more), employment status (employed, unemployed, not in labour force), age (continuous in years), sex (male, female), race/ethnicity (non-Hispanic white, non-Hispanic black, non-Hispanic other, Hispanic), nativity (U.S born, foreign-born), marital status (married, never married, divorced or separated, widowed), health insurance (at least private insurance, public insurance only, uninsured), region of residence (Northeast, Midwest, South, West) and survey year (dummies).

### Statistical analyses

Analyses were conducted in Stata se/15.1, and all adjusted for complex survey design. First, we conducted descriptive analyses to summarise demographic characteristics of the full study sample, respondents with disabilities and respondents without disabilities. Next, we estimated the association between income and psychological distress by conducting a series of linear regression models. The A series base models investigated the association between income and psychological distress for the full study sample, first without any interactions and then fully interacted by disability status (i.e. all predictor variables interacted with disability). The A series models adjusted for the entire list of covariates except food security. We then estimated the B series models, which assessed whether access to food helps explain observed associations by adding level of food security to the A series models. Post-estimation adjusted Wald tests were conducted to test whether food security explained a significant proportion of the association between income and psychological distress for each sample and whether the proportion explained differed by disability status.

## Results

### Sample characteristics

Weighted bivariate descriptive analyses (see Table 1) indicated that, in the general population, adults report an average psychological distress score of 2·56 points on the K6 Scale (sd = 2·62). Most (89·27 %) live in households that are food secure and have incomes at least two times greater than the federal poverty threshold (68·56 %). People with disabilities, on average, reported 2·5 times higher psychological distress levels (mean = 5·03 K6 units, sd = 4·11) than people without disabilities (mean = 2·04 K6 units, sd = 2·09). People with disabilities were also at a disproportionate socio-economic and material disadvantage and were more likely to be members of other marginalised groups. Compared to people without disabilities, those with disabilities were two times more likely to live in poverty (11·46 *v*. 22·43 %), two times more likely to report low food security (10·50 *v*. 5·10 %) and nearly three times more likely to report very low food security (11·86 *v*. 3·17 %). People with disabilities also tended to be less educated and were more likely to be unemployed or not in the labour force; older; female; non-Hispanic black; divorced, separated, or widowed; born in the USA; receiving only public health insurance; and residents of the South than those without disabilities. All observed bivariate differences in sample characteristics between adults with and without disabilities were statistically significant at the α = 0·01 level.

**Table 1:** Weighted characteristics of US adults included in a study of income, food security and psychological distress, by disability, National Health Interview Survey, 2011–2017*

	Full sample (*n* 102 598)		No disability (*n* 81 646)		Disability (*n* 20 952)	
	Mean or %	sd	Mean or %	sd	Mean or %	sd
Psychological distress	2·56 %	2·62	2·04 %	2·09	5·03 %	4·11
Income to poverty ratio (IPR)						
IPR ≥4	39·70		43·13		23·51	
IPR 2–<4	28·86		29·00		28·21	
IPR 1–<2	18·06		16·41		25·85	
IPR < 1	13·37		11·46		22·43	
Food security						
Secure	89·27		91·72		77·64	
Low	6·05		5·10		10·50	
Very low	4·69		3·17		11·86	
Education						
≥ Bachelor’s degree	30·80		33·73		16·94	
Some college	31·07		31·36		29·70	
High school/GED	25·13		23·98		30·58	
< High school	12·99		10·92		22·78	
Employment						
Employed	62·40		69·94		26·74	
Unemployed	4·93		5·06		4·32	
Not in labour force	32·67		25·00		68·94	
Age	46·40 %	11·86	43·92 %	10·94	58·11 %	12·99
Female	51·38		51·13		52·53	
Race/ethnicity						
White	65·82		64·88		70·26	
Black	11·30		11·08		12·34	
Hispanic	15·49		16·26		11·85	
Other	7·39		7·78		5·54	
Foreign-born	18·06		19·30		12·20	
Marital status						
Married	53·57		55·34		45·22	
Never married	27·11		28·74		19·39	
Divorced/separated	13·62		12·17		20·45	
Widowed	5·70		3·74		14·95	
Insurance						
Private	64·52		69·18		42·44	
Public	22·08		16·54		48·30	
None	13·4		14·28		9·26	
Region						
Northeast	17·39		17·64		16·21	
Midwest	22·64		22·60		22·81	
South	36·57		35·87		39·90	
West	23·40		23·88		21·08	

GED, general educational development.

* *T* and χ^2^ tests indicated that all characteristics were statistically different between those with and without disabilities at the 5 % level.

### Linear regression results

Results for the A and B series models are presented in Table 2. Model 1A explored the association between income and psychological distress among the general population. Models 2A and 3A reflect results from the model that was fully interacted with disability status. For clarity of presentation, we present coefficients from models stratified by disability, which are mathematically equivalent to the total effect of each coefficient in the fully interacted model (i.e. main coefficient added to the coefficient for the interaction term) for those without disabilities (Model 2A) and those with disabilities (Model 3A). Differences in the magnitude of associations by disability status reflect coefficients of the corresponding interaction terms.

**Table 2:** Linear regression results for psychological distress among US adults, National Health Interview Survey, 2011–2017†

	Full sample				No disability				Disability			
	Model 1A		Model 1B		Model 2A		Model 2B		Model 3A		Model 3B	
	Coefficient	95 % CI	Coefficient	95 % CI	Coefficient	95 % CI	Coefficient	95 % CI	Coefficient	95 % CI	Coefficient	95 % CI
Income (ref: IPR ≥4)												
IPR 2–<4	0·48*	0·41, 0·56	0·38*	0·30, 0·46	0·34*	0·26, 0·41	0·27*	0·19, 0·35	0·79*	0·54, 1·04	0·61*	0·36, 0·86
IPR 1–<2	1·00*	0·90, 1·11	0·62*	0·51, 0·72	0·65*	0·55, 0·75	0·42*	0·32, 0·52	1·51*	1·20, 1·81	0·91*	0·61, 1·21
IPR < 1	1·45*	1·32, 1·58	0·81*	0·69, 0·94	0·81*	0·69, 0·93	0·47*	0·35, 0·58	2·10*	1·74, 2·46	1·14*	0·78, 1·50
Food security (ref: secure)												
Low			1·88*	1·72, 2·04			1·49*	1·30, 1·66			1·68*	1·34, 2·03
Very low			4·04*	3·82, 4·26			2·76*	2·50, 3·02			4·11*	3·73, 4·49
Education (ref: ≥ bachelor’s degree)												
Some college	0·25*	0·18, 0·32	0·19*	0·12, 0·26	0·13*	0·06, 0·20	0·09*	0·03, 0·16	0·42*	018, 0·67	0·37*	0·13, 0·61
HS/GED	0·27*	0·18, 0·36	0·19*	0·11, 0·28	0·09*	0·01, 0·16	0·03	−0·04, 0·11	0·56*	0·28, 0·84	0·53*	0·26, 0·81
< HS	0·60*	0·47, 0·72	0·42*	0·30, 0·55	0·24*	0·11, 0·37	0·14*	0·01, 0·27	0·69*	0·39, 1·00	058*	0·28, 0·89
Employment (ref: employed)												
Unemployed	1·25*	1·06, 1·44	0·99*	0·80, 1·18	1·05*	0·86, 1·24	0·90*	0·72, 1·08	2·02*	1·43, 2·61	1·52*	0·93, 2·11
NILF	1·05*	0·95, 1·15	0·96*	0·87, 1·06	0·17*	0·09, 0·26	0·18*	0·09, 0·27	1·98*	1·69, 2·27	1·86*	1·58, 2·1
Constant	1·37*	1·25, 1·50	1·33*	1·21, 1·45	1·26*	1·15, 1·37	1·22*	1·11, 1·33	3·25*	2·82, 3·68	3·06*	2·64, 3·47

IPR, income to poverty ratio; GED, general educational development; HS, high school; NILF, not in labour force; ref, reference category.

* Indicates *P* < 0·05.

† All models controlled for gender, age, race/ethnicity, nativity, marital status, insurance, region and survey year.

**Fig. 2:**
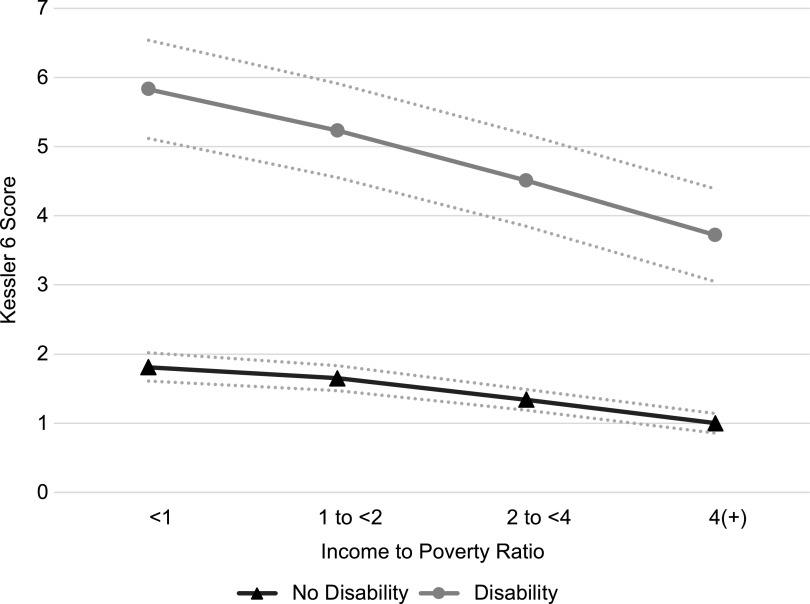
Predicted psychological distress among US adults by income and disability status, National Health Interview Survey, 2011–2017. Predicted levels were calculated based on results from Models 2A and 3A, holding all covariate values at their sample means. Dashed lines indicate 95 % CI

Consistent with extant evidence^(3)^, psychological distress increased as income decreased in a stepwise fashion, regardless of disability status. Compared to adults with incomes at least four times the poverty threshold, those in the general population with incomes 2 to <4, 1 to <2 and <1 times the poverty threshold scored 0·48 (95 % CI (0·41, 0·56)), 1·00 (95 % CI (0·90, 1·11)) and 1·45 (95 % CI (1·32, 1·58)) points higher on the K6 Scale, respectively. As observed in previously published research^(3)^, the coefficients for interaction terms between IPR and disability status indicated that the inverse association between income and psychological distress was stronger for those with disabilities than for those without disabilities at the α = 0·01 level. For instance, compared to those in the highest income category, individuals without disabilities living below the poverty threshold scored 0·81 (95 % CI (0·69, 0·93)) points higher on the K6 Scale. In comparison, individuals with disabilities living below the poverty threshold reported a difference in K6 score that was an additional 1·30 (95 % CI (0·93, 1·67)) points larger. Based on the results from Models 2A and 3A, predicted levels of psychological distress by IPR and disability status are presented in Fig. 2. Consistent with a more severe impact of financial hardship on mental health for those with disabilities, the disparity in psychological distress between people with and without disabilities widens as income decreases.

**Fig. 3:**
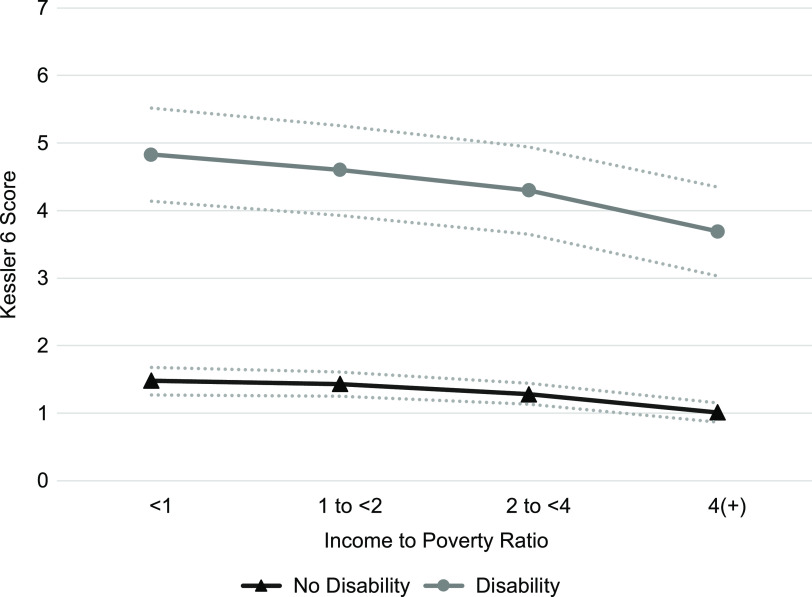
Predicted psychological distress among US adults by income and disability status after controlling for food security, National Health Interview Survey, 2011–2017. Predicted levels were calculated based on results from Models 2B and 3B, holding all covariate values at their sample means. Dashed lines indicate 95 % CI

To assess whether insufficient access to food helps explain the observed income-psychological distress relationship, food security was added in the B series models. A significant association between food insecurity and psychological distress and a reduction in the estimated income–psychological distress association were observed. Post-estimation adjusted Wald tests indicated that, regardless of disability status, food insecurity explained a statistically significant proportion of the detrimental relationship between low income and psychological distress. In the general population, accounting for food security reduced the income disparity between those living in poverty and those in the highest income category by 0·63 (95 % CI (0·59, 0·68)) points on the K6 Scale (or 44 %). Post-estimation adjusted Wald tests of interaction terms between IPR and disability status demonstrated that the proportion of the adverse association between income and psychological distress explained by food insecurity statistically differed by disability status. That is, accounting for food insecurity reduced the steepness of the income-psychological distress gradient significantly more for adults with disabilities than those without disabilities at the α = 0·01 level. For instance, among individuals with disabilities, accounting for food insecurity reduced the mental health disparity between those in poverty and those in the highest income category by 0·96 points (95 % CI (0·83, 1·10)) on the K6 Scale (or 46 %). In contrast, food insecurity only reduced the income disparity for those without disabilities by 0·34 points (95 % CI (0·31, 0·38)) (or 42 %). Consequently, the K6 poverty disparity between those with and without disabilities was reduced by a statistically significant proportion, 48 % or 0·62 points (95 % CI (0·49, 0·76)). Figure 3 presents predicted levels of psychological distress across income levels for people with and without disabilities based on the results from Models 2B and 3B. Compared to Fig. 2, Fig. 3 illustrates that after accounting for food insecurity, the steepness of the income-psychological distress gradient observed among people with disabilities more closely resembles the gradient for those without disabilities (i.e. the difference in the magnitude of the association between income and psychological distress for those with and without disabilities, after accounting for food insecurity, was reduced).

Independent of income, education and employment, the association between food security and psychological distress also followed a consistent, stepwise, inverse association (Models 1B, 2B and 3B). Compared to the food secure, those in the general population who experienced low and very low food security scored 1·88 (95 % CI (1·72, 2·04)) and 4·04 (95 % CI (3·82, 4·26)) points higher on the K6 Scale, respectively. Individuals with and without disabilities experiencing low food security scored 1·68 (95 % CI (1·34, 2·03)) and 1·49 (95 % CI (1·30, 1·66)) points higher on the K6 Scale than those who were food secure, respectively. The difference in the increase in K6 score between those with and without disabilities for low food security was not statistically significant. Very low food security, however, predicted psychological distress more strongly among people with disabilities than among those without disabilities. Compared to the food secure, individuals without disabilities experiencing very low food security scored 2·76 (95 % CI (2·51, 3·02)) points higher on the K6 Scale. Among individuals with disabilities, those reporting very low food security scored 4·11 (95 % CI (3·74, 4·49)) points higher than the food secure, a difference that was 1·35 (95 % CI (0·89, 1·81)) points larger than observed among those without disabilities.

## Discussion

To the best of our knowledge, this study is the first to investigate whether food insecurity helps explain the deleterious association between low income and psychological distress and whether its role differs by disability status in a nationally representative sample. Consistent with extant evidence, we observed an inverse association between income and poor mental health for the general population as well as for subgroups with and without disabilities^(1–3)^. Further, this adverse association was amplified for individuals with disabilities, echoing results of previous research^(3)^. That is, as income decreased, psychological distress increased more severely among people with disabilities than those without disabilities. Accounting for food insecurity reduced the compounded burden of poverty on mental health for people with disabilities by nearly half. These findings are consistent with the ICF framework^(21)^, which posits that social and environmental circumstances, including poverty and food insecurity, interact with an individual’s underlying medical condition to exacerbate disability and worsen health outcomes.

Results suggest that food insecurity plays a more important role in shaping patterns of poor mental health among people with disabilities than among those without disabilities, predicting psychological distress more strongly and explaining more of the income–psychological distress gradient. Among adults with disabilities, accounting for food insecurity reduced the disparity in K6 score for those living in poverty compared to the highest income category by nearly one full point, compared with only a third of a point for those without disabilities. Independent of income, very low food insecurity was further associated with an approximately four-point increase in psychological distress scores on the K6 Scale for those with disabilities, compared to less than 3 points for those without disabilities. To put these estimates into perspective, one study found that an increase of as little as two points in K6 score predicted increased odds of dying at an early age^(5)^.

In addition to these findings, supplemental analyses conducted using consistent measures and model specifications (results available upon request) demonstrated that the magnitude of the difference in the adverse association between food insecurity and mental health by disability status was larger than what has been reported by characteristics such as age, gender and region^(17,18,36)^. Food insecurity also explained a greater proportion of the difference in the magnitude of the income-mental health association by disability status than by these demographic characteristics. Our findings, however, differ somewhat from those of Brucker^(26)^. While results from both studies indicated that food insecurity increased the odds of reporting poor mental health, Brucker^(26)^ did not observe a difference in the strength of this relationship by disability status. Whereas Brucker’s^(26)^ study analysed one year of NHIS data and utilised binary indicators for both food insecurity and poor mental health, our study pooled seven years of NHIS data (i.e. a sample size that was nearly eight times larger) and used finer gradations of indicators (i.e. three levels of food security and a continuous measure of psychological distress), both of which may have enhanced our ability to detect differences in the magnitude of associations examined^(5,35)^.

There are several reasons why food insecurity may explain more of the income–psychological distress gradient and be more predictive of psychological distress, net of income, among people with disabilities. First, even at the same level of income, people with disabilities are more likely to experience food insecurity than individuals without disabilities^(22,23)^. To be food secure, people with work-related disabilities require an income at least 2·7 times higher than individuals without disabilities^(23)^. Those with disabilities may require higher-cost foods to manage their health conditions and spend more to access the same foods as those without disabilities. To manage their underlying health conditions, many individuals with disabilities must purchase higher-cost, higher-quality and specialty dietary items^(25)^. Those with difficulty travelling to stores, shopping for groceries and preparing food may have to spend more to purchase already prepared foods or have them delivered to their homes^(25)^.

Food insecure households also practice many cost-saving strategies that can directly harm health and exacerbate existing medical conditions, including skipping, delaying or taking less medication than prescribed; postponing or refusing needed medical care; failing to follow recommended diets for their health conditions or consuming lower-quality, higher-calorie foods^(37,38)^. ^[Bibr r37]^^[Bibr r37]^Compared to individuals without disabilities, those with disabilities have more competing demands on their limited financial resources and greater unmet needs for basic necessities such as medications; medical equipment, services and supplies; housing and transportation^(22,28)^. One in three adults with chronic illness cannot afford their medications, food or both^(37)^. Chronically ill adults experiencing very low food security have four times higher odds of underusing prescribed medications because of cost than those who are food secure^(37)^. Hence, people with disabilities may be constantly faced with making distressing choices between quality and quantity of food or healthcare and medication^(22,26)^.

Our findings are particularly concerning given that food insecurity seems to be more entrenched among those with disabilities. Although the prevalence of food insecurity decreased in the general population from 14·6 % in 2008 to 11·1 % in 2018, food insecurity among people with work-related disabilities remained constant at 33 %^(24)^. Further, many safety net programmes designed to improve food security are insufficient to meet the needs of people with disabilities^(25)^. Community gardening and free meal programmes have demonstrated little utility for improving food security among people with disabilities and are often inaccessible to participants with disabilities^(25)^. Hot meal programmes and food pantries often fail to consider transportation and mobility barriers, requiring consumers to travel to the site, use the services within a limited window of time and, in some cases, transport goods back home without assistance^(25)^.

Research demonstrates that even adult with disabilities who receive income assistance through Social Security Disability Insurance (SSDI) and Supplemental Security Income (SSI) have disproportionately high odds of experiencing food insecurity^(27)^. Evidence suggests that monetary support provided by these programmes is insufficient to meet the basic material needs of many people with disabilities. Nearly 45 % of adults who receive SSI and 20 % who receive SSDI live in households with total incomes below the federal poverty threshold^(39)^. Because these programmes, along with Medicaid and the Supplemental Nutrition Assistance Program, are means-tested and impose strict limits on assets, individuals with disabilities are additionally discouraged from building assets that could reduce the impact of short-term income losses and unexpected medical costs on food security^(22)^. Huang and colleagues^(22)^ found that, among people with disabilities, household assets such as a homeownership, savings and net worth are more protective against food insecurity than income. Thus, it is not surprising that, in addition to partially explaining the relationship between poverty and poor mental health, we found that very low food insecurity predicts psychological distress more strongly among adults with disabilities than those without disabilities independently of income.

### Limitations

Our findings should be interpreted in light of the study’s limitations. A scoping review of extant literature suggests that people with disabilities can be trapped in complex, multidirectional cycles of poverty, food insecurity and disability^(25)^. Because the NHIS is a cross-sectional survey, we were unable to draw inferences about the direction or causality of the relationships observed. Second, the NHIS sampling frame excludes people experiencing homelessness and those living in institutional settings. While institutionalised populations might be more likely to be food secure than those living in the community, they often have less control over the type, quality and quantity of food they consume^(25)^. As a consequence, our results may not be generalisable to these subgroups. Third, people with chronic conditions and disabilities commonly underreport symptoms of poor mental health, scoring higher on social desirability bias measures than those without disabilities^(40,41)^. Finally, the K6 Scale only measures symptoms of psychological distress experienced in the last 30 d^(30)^. As a result, it may capture emotional responses to short-term stressors in addition to symptoms of longer-term psychological distress^(30)^.

## Conclusions

Results suggest that socio-economic and material disadvantages, including poverty and food insecurity, may be stronger determinants of poor mental health among people with disabilities than those without disabilities. Consistent with extant evidence^(3)^, we observed a stronger detrimental association between poverty and psychological distress among people with disabilities than among those without disabilities. Food insecurity explained more of the income–psychological distress gradient among people with disabilities than those without disabilities and predicted psychological distress more strongly independent of income, education and employment status. These findings are particularly troubling given that people with disabilities, who are a large and growing population, are two times more likely to live in poverty and three times more likely to report very low food security than people without disabilities. Reducing food insecurity may help reduce long-entrenched mental health disparities experienced by people with disabilities.
